# College and Hospital Notes

**Published:** 1909-07

**Authors:** 


### COLLEGE AND HOSPITAL NOTES

---

The Harrington Hospital for Children, on Goodrich Street, opposite the Buffalo General Hospital, was opened Thursday afternoon, May 27, 1909. The exercises were under the direction of the trustees of the Buffalo General Hospital. The Journal acknowledges the courteous invitation of the trustees to attend the ceremonies.

---

LeRoy has excellent prospects for a hospital and physicians have suggested purchasing the site and building of LeRoy old high school on East Main Street. The property is owned by the school district and can be bought for $10,000. The site is an excellent one and the buildings are well adapted for use as a hospital on account of the large, airy and well lighted rooms. A number of citizens have expressed their willingness to aid in the work.

